# Validation of the Korean version of the Utrecht Grief Rumination Scale and its relationship with COVID‐related hypochondriasis among healthcare workers who witnessed patient deaths

**DOI:** 10.1002/brb3.3203

**Published:** 2023-09-04

**Authors:** Jeong Hye Kim, Seockhoon Chung

**Affiliations:** ^1^ Department of Clinical Nursing University of Ulsan Seoul South Korea; ^2^ Department of Psychiatry, ASAN Medical Center University of Ulsan College of Medicine Seoul South Korea

**Keywords:** anxiety, death, grief, health personnel, hypochondriasis

## Abstract

**Introduction:**

This study aimed to examine the reliability and validity of the Korean version of the Utrecht Grief Rumination Scale (UGRS) among healthcare workers who witnessed patient deaths. We also examined whether grief rumination may impact the cognitive‐behavioral model of hypochondriasis.

**Methods:**

This study was conducted via an anonymous online survey targeting healthcare workers who had worked at a tertiary hospital and had witnessed patient deaths over the previous 2 years. Demographic data and responses to the UGRS, the Pandemic Grief Scale (PGS) for healthcare workers, the Stress and Anxiety to Viral Epidemic‐9 (SAVE‐9), the Obsession with COVID‐19 Scale (OCS), and the Coronavirus Reassurance‐Seeking Behaviors Scale (CRBS) were collected by requesting participants to recall their emotional state during the 2 weeks after witnessing a patient's death.

**Results:**

The Korean version of the UGRS is reliable (Cronbach's alpha = 0.941) and valid (comparative fit index = 0.920, Tucker–Lewis index = 0.900, root‐mean‐square‐error of approximation = 0.102, standardized root‐mean‐square residual = 0.050) for measuring grief rumination in healthcare workers. The OCS was predicted by CRBS (*β* = 0.19, *p* < 0.001), SAVE‐9 (*β* = 0.45, *p* < 0.001), UGRS (*β* = 0.16, *p* = 0.010), and PGS (*β* = 0.16, *p* = 0.010, adjusted *R* ^2^ = 0.49, *F* = 52.9, *p* < 0.001). In mediation analysis, grief rumination directly influenced coronavirus preoccupation; the relationship was mediated by viral anxiety and coronavirus reassurance‐seeking behavior.

**Conclusion:**

Grief rumination of healthcare workers who witnessed patient death requires further exploration as it may influence hypochondriacal responses.

## Graphical abstract

Grief rumination of healthcare workers directly influenced their preoccupation with the coronavirus. The relationship was mediated by viral anxiety and coronavirus assurance‐seeking behavior.

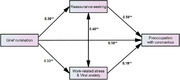

## 1 INTRODUCTION

The COVID‐19 (coronavirus disease) pandemic has caused many changes and difficulties for the world, with the World Health Organization reporting 763 million confirmed COVID‐19 cases and 6.9 million fatalities. Many people worldwide are grieving the loss of loved ones due to COVID‐19, such as family, friends, and colleagues, with the frequency of grief‐related problems reported to be much higher during the pandemic than before its outbreak (Kustanti et al., 2023). The clinically significant symptoms of anxiety and depression throughout the COVID‐19 pandemic have been reported (Guerrini et al., 2021). Due to social distancing and restrictions enacted on hospital visits due to concerns over infection, end‐of‐life care, funerals, and burials could not be performed as before the pandemic; consequently, healthcare workers did not have enough time for the mourning process (Wallace et al., 2020). An inappropriate or incomplete grieving process can lead to maladaptive symptoms, resulting in complicated grief (Wallace et al., 2020). While caring for patients with COVID‐19, nurses were frequently exposed to patient deaths, and more than half of them experienced complicated grief. Grief is a normal process that helps individuals adapt to loss. If grief is not fully resolved, it may have an adverse impact on physical and mental health, eventually resulting in burnout (Rahmani et al., 2023).

Furthermore, healthcare workers encounter numerous challenges, given their responsibility for caring for infected patients during difficult times. Additionally, they frequently witnessed the deaths of patients they cared for throughout the pandemic (Rahmani et al., 2023). There have been reported cases of psychological distress among healthcare workers due to their challenging work, lack of access to personal preventive equipment, lack of situation‐specific training, and fear of infecting family members and coworkers (Das et al., 2021).

Normal grief can cause severe psychological reactions such as anger and shock; however, within 1 year after the death of a loved one or acquaintance, grief typically develops into an integrated stage and does not require special therapeutic intervention. Although most people adequately adjust to the grief associated with loss, for some, such grief develops into psychological distress (De Stefano et al., 2021). Thus, the unexpected and violent loss of a loved one can negatively impact mental health and the grieving process, resulting in mental illnesses such as complicated grief, major depressive disorder, and post‐traumatic stress disorder. Additionally, active intervention is needed because mental health problems occur more often, and recovery in the case of an unexpected, sudden, and violent loss takes longer than that of a loss due to natural causes (Kristensen et al., 2012). During the COVID‐19 pandemic, healthcare workers witnessed the deaths of numerous patients, experiencing complicated grief due to unexpected and agonizing loss. This phenomenon persisted after the pandemic had ended (Rahmani et al., 2023). Repetitive negative thoughts such as rumination and worry are risk factors for severe and persistent complicated grief due to loss (Eisma et al., 2022). There are two kinds of rumination: depressive rumination and grief rumination. Grief rumination comprises the significance of loss and general feelings associated with the experience, whereas depressive rumination concentrates on depressive feelings and symptoms. Grief rumination is not limited to depressed feelings but extends to the negative emotions related to loss in general, often leading to other types of rumination. Therefore, grief rumination is more likely to occur than the depressive rumination (Eisma et al., 2014). The Utrecht Grief Rumination Scale (UGRS) is a system developed to evaluate grief‐specific rumination, which addresses repetitive and self‐focused thoughts about the causes and consequences of loss and related negative feelings. Rumination and worry are related to depression and complicated grief (Eisma et al., 2020).

Repetitive negative thoughts following a loss can significantly affect mental health. During the COVID‐19 pandemic, healthcare workers expressed concerns and fears that they would become infected and spread the coronavirus to their families (Gray et al., 2021). To prevent COVID‐19 infection, several safety behaviors were implemented in public places, including frequent hand washing, physical distancing, and wearing masks or face coverings. Most people felt anxious and threatened throughout this period, consequently engaging in safety behaviors. For some individuals, a pandemic may cause excessive anxiety and obsessive‐compulsive behaviors such as frequent hand washing and hand sanitizer use, both of which increased throughout the course of the pandemic. Healthcare workers resort to negative psychological strategies, including hypochondriasis, when placed under significant pressure and stress; notably, moderate levels of hypochondria have been associated with the COVID‐19 pandemic (Yusefi et al., 2021).

Considering these circumstances, we hypothesized that the grief rumination of healthcare workers might influence the perpetuation of hypochondriacal reactions when facing coronavirus‐related events or when caring for patients infected with the virus. This study examined the reliability and validity of the Korean version of the UGRS among healthcare workers who have been exposed to patient deaths. Additionally, we examined whether grief rumination among healthcare workers played a role in the cognitive‐behavioral model of hypochondriasis. We posit that grief rumination in healthcare workers is (1) positively correlated with viral anxiety, (2) positively correlated with coronavirus‐related reassurance‐seeking behaviors, (3) positively correlated with the preoccupation with coronavirus, and (4) an influence on the preoccupation with coronavirus, which is mediated by viral anxiety and reassurance‐seeking behaviors.

## 2 METHODS

### 2.1 Participants and procedure

This study used data obtained from a previous study conducted through anonymous online surveying of healthcare workers at a tertiary hospital from June 2 to 10, 2022 (D. Lee et al., 2022). We recruited healthcare workers (doctors and nurses) who experienced the deaths of patients over the preceding 2‐year period. A survey link with enrollment information was posted on the hospital's intranet, allowing for voluntary participation in this survey. Participants were provided a reward of a five dollar‐gift coupon for participating. We obtained demographic information of participants regarding age, sex, marital status, years of employment, and whether they were required to do shift work. Questions regarding COVID‐19 were also included in the survey, including those inquiring about experiences of being infected, being quarantined, or getting vaccinated. The survey form was developed in accordance with the Checklist for Reporting Results of Internet E‐Surveys (Eysenbach, 2004).

We estimated a sample size based on the Central Limit Theorem, resulting in 30 samples for every 10 cells (doctors vs. nursing professionals X five age groups; Cohen, 1988). However, we could only collect feedback from 263 of the 293 respondents, excluding 26 participants who had not experienced patient deaths in the period under consideration.

### 2.2 Survey scales

We requested participants to recall their emotional state during the 2 weeks immediately after witnessing a patient's death and to respond to each rating scale based on the experience.

#### 2.2.1 UGRS

The UGRS measures an individual's rumination on grief (Eisma et al., 2014). It consists of 15 items, each of which is rated from 1 (*never*) to 5 (*very often*) on a 5‐point scale. A higher total score (ranging from 15 to 75) indicates a high level of grief rumination. Previously, it has been reported to have five components: Meaning (items 1, 2, and 15, regarding the meaning and consequences of the death), Relationships (items 3, 9, and 14, concerning reactions from others), Counterfactuals (items 4, 8, and 10, relating to counterfactual thought concerning the events leading to the loss), Injustice (items 5, 11, and 12 pertaining to the injustice of the loss), and Reactions (items 6, 7, and 13, addressing the emotional reactions to the loss; Sveen et al., 2019). The Korean version of the UGRS was developed via translation and back‐translation methods (Supplementary Information). First, two bilingual experts translated the English version of the UGRS into two Korean versions. Second, another two bilingual experts re‐translated the combined translated Korean version of the UGRS into English. Finally, another bilingual expert compared the original English version and two reversed translated English versions of the UGRS and checked for subtle variations. Permission was obtained from the developer of the UGRS to translate the scale.

#### 2.2.2 Pandemic Grief Scale (PGS) for healthcare workers

The PGS is a rating scale that can be used to measure grief reactions to loss experienced throughout the pandemic (S. A. Lee & Neimeyer, 2022). An alternative healthcare worker‐specific PGS has also been developed. In this study, we applied the Korean version of the PGS for healthcare workers (J. H. Kim et al., 2023), which consists of five items rated on a 4‐point scale ranging from 0 (*not at all*) to 3 (*nearly every day*). A higher total PGS score indicates a higher level of dysfunctional grief as a result of having experienced loss throughout the COVID‐19 pandemic. The Cronbach's alpha for this sample was 0.842.

#### 2.2.3 Stress and Anxiety to Viral Epidemic‐9 items (SAVE‐9)

The SAVE‐9 is a rating scale that can measure work‐related stress and anxiety in response to viral epidemics and was specifically created with healthcare workers in mind (Chung et al., 2021). It consists of nine items that can be rated on a 5‐point scale, ranging from 0 (*never*) to 4 (*always*). A higher total score indicates a higher level of work‐related stress and viral anxiety experienced by healthcare workers. This study used the original Korean version, and in this sample, Cronbach's alpha was 0.873.

#### 2.2.4 Obsession with COVID‐19 Scale (OCS)

The OCS is a scale that can assess an individual's preoccupation with the coronavirus (S. A. Lee, 2020). The OCS consists of four items and can be rated on a 5‐point scale ranging from 0 (not at all) to 4 (nearly every day over the last 2 weeks). A higher total score indicates a greater level of preoccupation with the coronavirus. The Korean version of the OCS was applied in this study (Choi et al., 2022), and Cronbach's alpha among this sample is 0.832.

#### 2.2.5 Coronavirus Reassurance‐Seeking Behaviors Scale (CRBS)

The CRBS is a scale that can assess reassurance‐seeking behavior in an individual concerning the coronavirus pandemic (S. A. Lee et al., 2020). The CRBS consists of five items that are rated on a 5‐point scale ranging from 0 (*not at all*) to 4 (*nearly every day over the last 2 weeks*). A higher total score indicates a higher tendency for reassurance‐seeking. In this investigation, we used the Korean version of the CRBS (C. Kim et al., 2022), with a Cronbach's alpha of 0.900 among this sample.

### 2.3 Ethical considerations

The study protocol was approved by the Institutional Review Board of ASAN Medical Center (Approval no. 2022‐0740), and the need for obtaining written informed consent was waived.

### 2.4 Statistical analysis

First, we examined the reliability and validity of the UGRS among healthcare workers who witnessed the death of patients they attended. Before the confirmatory factor analysis (CFA), the normality of all 15 items was checked based on skewness ranging within ± 2 and a kurtosis ranging within ± 7 (Byrne, 2001). A Kaiser–Meyer–Olkin (KMO) measure and Bartlett's test of sphericity were used to check sampling adequacy and data suitability. The CFA examined the structure validity of the five‐factor model of the UGRS. We clustered all 15 items into five subcategories; Meaning (items 1, 2, and 15), Relationships (items 3, 9, and 14), Counterfactuals (items 4, 8, and 10), Injustice (items 5, 11, and 12), and Reactions (items 6, 7, and 13; Sveen et al., 2019). A satisfactory model fit was defined by a standardized root‐mean‐square residual (SRMR) value ≤ 0.05, a root‐mean‐square‐error of approximation (RMSEA) value ≤ 0.10, and comparative fit index (CFI) and Tucker–Lewis index (TLI) values ≥ 0.90 (Brown, 2006; Byrne, 2001). Internal consistency reliability was tested based on Cronbach's alpha. Pearson's correlation coefficients were calculated to check the convergent validity of the UGRS with other rating scales.

Second, Pearson's correlation coefficients were calculated between age and symptom rating scales. A linear regression analysis with the enter method was conducted to identify which variables could be expected to cause preoccupation with the coronavirus pandemic. Mediation analysis was used to test the feasibility of the cognitive‐behavioral model of COVID‐related hypochondriasis among healthcare workers who witnessed patient deaths. Additionally, bootstrapping of all 2000 resamples was conducted to explore whether the influence of grief rumination on the preoccupation with coronavirus may be mediated by viral anxiety and reassurance‐seeking behavior.

Demographic data and scale scores were presented as mean ± standard deviation. The significance level was defined as two‐tailed with a threshold of *p* < 0.05. SPSS version 21.0, AMOS version 27 for Windows (IBM Corp.) and JASP 0.17.1.10. were used to perform the statistical analysis.

## 3 RESULTS

### 3.1 Reliability and validity of the Korean version of the UGRS

Demographic characteristics and symptom rating scale scores are presented in Table 1. Normality was assumed based on the skewness (1.40−2.18) and kurtosis (2.38−5.78). The sampling was adequate, and the data were suitable for conducting factor analysis based on the KMO value (0.930) and Bartlett's test of sphericity (*p* < 0.001). CFA results demonstrated a good fit for the five‐factor model of the UGRS among healthcare workers who witnessed patient deaths (CFI = 0.920, TLI = 0.900, RMSEA = 0.102, SRMR = 0.050). Factor loadings of each item in each subcategory ranged between 0.40 and 0.77 (Table 2). The Korean version of the UGRS had good internal consistency in terms of reliability (Cronbach's alpha = 0.941) and demonstrated good convergent validity with the PGS (*r* = 0.68, *p* < 0.001), SAVE‐9 (*r* = 0.33, *p* < 0.001), CRBS (*r* = 0.39, *p* < 0.001), and OCS (*r* = 0.47, *p* < 0.001; Table 3).

**TABLE 1:** Demographic and clinical characteristics of participants (*N* = 267).

Variables	*n* (%) M ± SD
Gender (women)	210 (78.7)
Profession	
Doctors	91 (34.1)
Nurses	176 (65.9)
Age	31.4 ± 5.4
Years of employment	6.6 ± 5.4
**Marital status** ^a^	
Single	168 (63.4)
Married, without kids	35 (13.2)
Married, with kids	62 (23.4)
**Are you a shift worker? (Yes)**	153 (57.3)
**Questions on COVID‐19**	
Are you taking care of COVID‐19‐infected patients? (Yes)	221 (100.0)
Did you experience being quarantined due to infection with COVID‐19? (Yes)	155 (58.1)
Did you experience being infected with COVID‐19? (Yes)	134 (50.2)
Did you get vaccinated? (Yes)	264 (98.9)
**Did you experience deaths of COVID‐19‐infected patients? (Yes)**	267 (100.0)
**Death of patients**	
Death was related to COVID‐19	62 (23.2)
Death was not related to COVID‐19	164 (61.4)
Unknown	41 (15.4)
**Psychiatric history**	
Have you experienced or received treatment for depression, anxiety, or insomnia? (Yes)	42 (15.7)
Now, do you think you are depressed or anxious, or do you need help for your mood state? (Yes)	19 (7.1)
**Rating scales scores**	
Utrecht Grief Rumination Scale	24.9 ± 9.3 (15–75)
Pandemic Grief Scale for healthcare workers	1.3 ± 2.1 (0–15)
Stress and Anxiety to Viral Epidemic‐9 items	14.1 ± 5.2 (0–24)
Obsession with COVID‐19 Scale	3.5 ± 3.1 (0–16)
Coronavirus Reassurance‐seeking Behavior Scale	5.7 ± 4.3 (0–19)

Abbreviations: COVID‐19, coronavirus disease; M, mean; SD, standard deviation.

^a^ There were two missing values.

**TABLE 2:** Item properties of the UGRS among healthcare workers who witnessed patients’ death.

	Response scale					Descriptive						
Items. How frequently in the past month did you…	1	2	3	4	5	M	SD	Factor loading				
1. …think about the consequences that their death has for you	50.2	44.2	2.2	2.2	1.1	1.6	0.7	0.59				
2. …analyze what the personal meaning of the loss is for you	28.8	61.0	6.4	1.9	1.9	1.9	0.8	0.55				
3. …question whether you receive the right support from family members	60.3	31.3	4.5	3.0	1.1	1.5	0.8		0.40			
4. …analyze whether you could have prevented their death	34.1	52.4	7.1	3.4	3.0	1.9	0.9			0.75		
5. …ask yourself why you deserved this loss	53.2	39.3	4.1	1.9	1.5	1.6	0.8				0.65	
6. …try to analyze your feelings about this loss precisely	52.8	40.1	3.0	2.2	1.9	1.6	0.8					0.73
7. …ask yourself whether you react normally to this loss	51.3	39.7	4.9	2.6	1.5	1.6	0.8					0.71
8. …ask yourself whether their death could have been prevented if the circumstances had been different	46.1	41.6	5.6	4.1	2.6	1.8	0.9			0.88		
9. …ask yourself whether you get adequate support from friends and acquaintances	43.1	45.3	6.0	4.5	1.1	1.8	0.8		0.60			
10. …ask yourself whether their death could have been prevented if others had acted differently	54.7	35.6	4.1	3.4	2.2	1.6	0.9			0.77		
11. …wonder why this had to happen to you and not someone else	64.0	29.2	3.0	2.6	1.1	1.5	0.8				0.53	
12. …think about the unfairness of this loss	62.9	30.3	3.0	2.2	1.5	1.5	0.8				0.60	
13. …try to understand your feelings about the loss	44.6	43.8	5.2	4.1	2.2	1.8	0.9					0.70
14. …think how you would like other people to react to your loss	49.8	38.6	6.4	2.6	2.6	1.7	0.9		0.77			
15. …think how your life has changed because of their death	50.2	40.1	6.4	1.5	1.9	1.6	0.8	0.70				

*Note*: 1 = never, 2 = sometimes, 3 = regularly, 4 = often, 5 = very often.

Abbreviations: M, mean; SD, standard deviation; UGRS, Utrecht Grief Rumination Scale.

**TABLE 3:** Correlation coefficients of each variable among healthcare workers who witnessed patient's death (*N* = 267).

Variables	Age	OCS	CRBS	SAVE‐9	UGRS
**OCS**	0.05		–		
**CRBS**	−0.004	0.77**			
**SAVE‐9**	−0.002	0.61**	0.61**		
**UGRS**	0.03	0.47**	0.39**	0.33**	
**PGS**	0.03	0.44**	0.38**	0.24**	0.68**

Abbreviations: CRBS, Coronavirus Reassurance‐seeking Behavior Scale; OCS, Obsession with COVID‐19 Scale; PGS, Pandemic Grief Scale for healthcare workers; SAVE‐9, Stress and Anxiety to Viral Epidemics‐9 items; UGRS, Utrecht Grief Rumination Scale.

*p < .05; **p < .01.

### 3.2 Grief rumination and COVID‐related hypochondriasis

**FIGURE 1:**
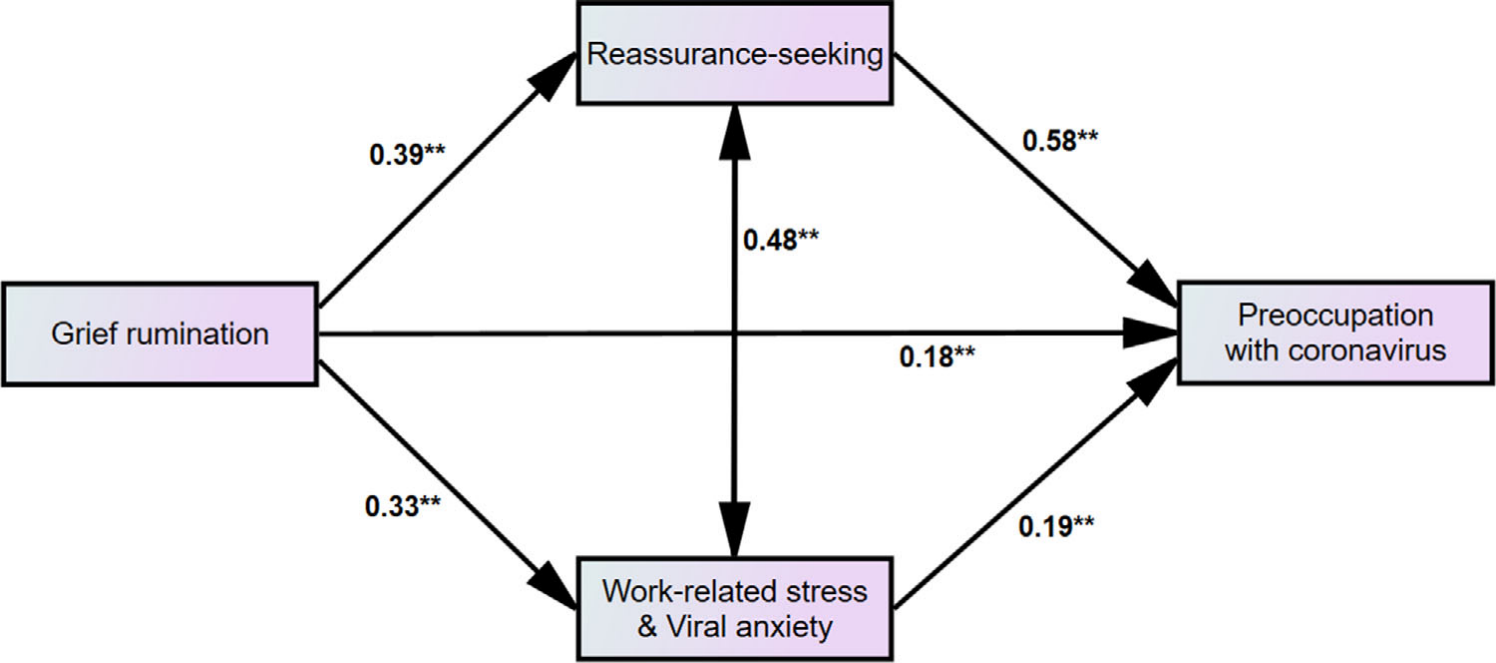
Mediation model showing the pathway from the effect of grief rumination (independent variables) on preoccupation with coronavirus (outcome) through work‐related stress and viral anxiety or coronavirus reassurance‐seeking behavior (mediator).

Linear regression analysis showed that the OCS could be predicted by the CRBS (*β* = 0.19, *p* < 0.001), SAVE‐9 (*β* = 0.45, *p* < 0.001), UGRS (*β* = 0.16, *p* = 0.010), and PGS scores (*β* = 0.16, *p* = 0.010, adjusted *R* ^2^ = 0.49, F = 52.9, *p* < 0.001; Table 4). The mediation analysis showed that the cognitive‐behavioral model of COVID‐related hypochondriasis was feasible in healthcare workers who have witnessed the death of patients (Supplementary Table S1). Regarding mediation analysis, we excluded the PGS in the final model. The mediation analysis results showed that grief rumination directly influenced preoccupation with coronavirus in participants and that the relationship was mediated through viral anxiety and coronavirus reassurance‐seeking behavior (Supplementary Table S2 and Figure 1).

**TABLE 4:** Linear regression analysis to explore variables which predict preoccupation with coronavirus among healthcare workers who witnessed patient deaths (*N* = 267).

Dependent variables	Included parameters	*β*	*p*	Adjusted *R* ^2^	*F*(*p*)
**OCS**	Age	0.09	0.062	0.49	*F* = 52.9(0.001)
	CRBS	0.19	< 0.001		
	SAVE‐9	0.45	< 0.001		
	UGRS	0.16	0.010		
	PGS	0.16	0.010		

Abbreviations: CRBS, Coronavirus Reassurance‐seeking Behavior Scale; OCS, Obsession with COVID‐19 Scale; PGS, Pandemic Grief Scale for healthcare workers; SAVE‐9, Stress and Anxiety to Viral Epidemics‐9 items; UGRS, Utrecht Grief Rumination Scale.

## 4 DISCUSSION

We observed that the Korean version of the UGRS was a reliable and valid rating scale for measuring the grief rumination experienced by healthcare workers who witnessed death during the COVID‐19 pandemic. Based on the CFA results, the scale demonstrated good construct validity as a five‐factor model and good internal consistency reliability. The Korean version of the UGRS showed good convergent validity with the preexisting valid rating scales used to measure an individual's psychological status related to COVID‐19, such as the SAVE‐9, CRBS, OCS, and PGS. Furthermore, the cognitive‐behavioral model of hypochondriasis was applicable among healthcare workers who have witnessed the deaths of patients throughout the COVID‐19 pandemic. Linear regression analysis showed that preoccupation with coronavirus was predicted by viral anxiety, reassurance‐seeking behavior, and grief responses. In the mediation analysis, we observed that the grief rumination of healthcare workers who witnessed patient deaths directly influenced their preoccupation with the coronavirus and that viral anxiety and coronavirus reassurance‐seeking behavior mediated this relationship.

We tested the construct validity of the Korean version of the UGRS as a five‐factor model in accordance with previous studies (Sveen et al., 2019). The construct validity was generally acceptable, and the factor loading value of item 3 “query whether you receive the right support from family members,” was the lowest (0.40) among all items in terms of the Relationships subcategory. The option of “never” as a response to item 3 was high (60.3%), compared to that of items 9 (43.1%) and 14 (49.8%). This may reflect that healthcare workers in Korean culture share their loss experience with friends or colleagues rather than family members. For individuals who do not work in places where the death of people is frequently experienced, such as in a hospital, experiencing the death of family members or friends can easily be communicated to the family. Unlike the general population, healthcare workers may find it challenging to share their experiences with family members due to their work environment.

The mean value of each item (1.5−1.9) in this study was relatively low, compared to a previous study in the general population (1.9‐4.2; Doering et al., 2018). The difference in grief responses may be due to cultural differences, the connection to a pandemic situation, or the specific sample of healthcare workers used. In this study, 90% of respondents answered each item with “never” or “sometimes.” This indicates that approximately 10% of participants responded that they think about grief on a regular basis, at the very least. Healthcare workers may experience a lower grief response than the general population; however, this could be the result of adaptation during the prolonged pandemic (which lasted for 3 years) rather than a lack of sadness over the death of patients.

In this study, the cognitive‐behavioral model of COVID‐related hypochondriasis was found to be applicable to healthcare workers who have witnessed patient deaths. Throughout the COVID‐19 pandemic, healthcare workers might have felt anxious concerning potential exposure to the viral infection both in the hospital environment and through their proximity to infected patients. Due to the fear of spreading the viral infection to their family members or friends, they may frequently check their temperatures and monitor themselves for symptoms associated with viral infections. In severe cases, they may become preoccupied with coronavirus symptoms.

When people ruminate on particular symptoms or events without taking action to alleviate them, they passively and repetitively concentrate on these thoughts (Stroebe et al., 2007). During this pandemic, the grief rumination of healthcare workers was characterized by strong attempts to reconcile the event with previous beliefs about the meaning or fairness of the world, as well as counterfactual thinking (Doering et al., 2018; Eisma et al., 2020). Their grief rumination may have caused them to become depressed or anxious about the events leading up to the loss (Eisma et al., 2020). Throughout the COVID‐19 pandemic, healthcare workers repeatedly reflected on patient deaths, even though these deaths were not directly caused by the coronavirus. This thought pattern may have led to viral anxiety and reassurance‐seeking behavior. In a hospital setting, especially given the chaotic nature of the COVID‐19 pandemic, it has been challenging to develop a plan for the psychological support of healthcare workers. In the face of patient deaths, healthcare workers could not alleviate their grief responses because healthcare professionals worldwide were striving to prevent similar tragedies from occurring (Hong et al., 2023). The findings of this study showed that a psychological support system, which can reduce the grief rumination of healthcare workers who witnessed the deaths of patients, is needed in the face of the COVID‐19 pandemic.

The study has some limitations. First, 61.4% of participants stated that the deaths of patients they had witnessed were not directly related to coronavirus. Healthcare workers in this pandemic were continuously concerned about the coronavirus, and their working environment could contribute to their distress, regardless of the reasons for death. Second, 78.7% of the participants in this sample were female. We previously observed that female participants had a high level of viral anxiety, which might lead to overestimation of the study results (Ahn et al., 2021). Third, the survey was conducted in a single private tertiary‐level hospital. Due to potential variations in viral transmission within hospitals and differences in the reasons or frequencies of deaths across different hospitals, the results cannot be generalized. Fourth, the study was conducted nearly 2 years after the onset of the pandemic. Therefore, the results may have been affected by how participants adapted to the situation. Finally, the small sample size is a limitation of this study. Further investigation is needed among larger samples and other participant groups.

## 5 CONCLUSION

We observed that the Korean version of the UGRS can be used to measure the grief rumination of healthcare workers who have witnessed the death of fellow healthcare workers with good reliability and validity. Additionally, we have also found that grief rumination of healthcare workers who witnessed patient deaths directly influenced their preoccupation with the coronavirus and that viral anxiety and coronavirus reassurance‐seeking behavior mediated this relationship. The UGRS scale can help healthcare workers assess their psychological status in relation to the pandemic and develop appropriate interventions to deal with a preoccupation with coronavirus, viral anxiety, and reassurance‐seeking behaviors that may arise during crises. Additionally, the cognitive‐behavioral model of hypochondriasis may help explain and guide the treatment of hypochondriasis among healthcare workers.

## AUTHOR CONTRIBUTIONS

**Jeong Hye Kim**: Conceptualization; methodology; writing—original draft; writing—review and editing. **Seockhoon Chung**: Conceptualization; data curation; formal analysis; investigation; methodology; project administration; writing—original draft; writing—review and editing.

### PEER REVIEW

The peer review history for this article is available at https://publons.com/publon/10.1002/brb3.3203.

## Supporting information

Supporting Information

## Data Availability

Data are available on request from the authors.
