# Supplementary material for: Validation of the Korean version of the Utrecht Grief Rumination Scale and its relationship with COVID‐related hypochondriasis among healthcare workers who witnessed patient deaths
Source: Brain Behav. 2023 Sep 4;13(10):e3203. doi: 10.1002/brb3.3203 (PMC10570490; doi:10.1002/brb3.3203)
Supplement: Supplementary file 1 — Supporting Information [file BRB3-13-e3203-s001.docx]

**Appendix. Supplementary file**

**Utrecht Grief Rumination Scale (Thought about loss)**

사람들은 사랑하는 사람이 세상을 떠난 후에 다양한 생각을 떠올리는 경우가 많습니다. 다음 질문은 지난 한 달 동안 다음과 같은 생각을 얼마나 자주 떠올렸는지 알아보기 위한 내용입니다.

**지난 한 달 동안 다음과 같은 생각을 얼마나 자주 하셨습니까?**

**한 번도 하지 않았다; 가끔 했다; 정기적으로 했다; 자주 했다; 매우 자주 했다**

1. 나는 고인의 죽음이 나에게 미칠 영향에 대해 생각해보았다.

2. 본인에게 상실이 의미하는 바가 무엇인지 분석해보았다.

3. 가족들이 나에게 충분한 힘이 되어주고 있는지 의문이 들었다.

4. 고인의 죽음을 막을 수 있었는지에 대해 분석해보았다.

5. 이런 상실을 겪어야 하는 이유가 있는지 스스로에게 물어보았다.

6. 이 상실에 대한 나의 감정을 정확히 분석하려고 노력해보았다.

7. 이 상실에 대한 반응이 정상적인지 스스로에게 물어보았다.

8. 상황이 달랐다면 고인의 죽음을 막을 수 있었는지 스스로에게 물어보았다.

9. 친구나 지인이 적절한 힘이 되어주고 있는지 스스로에게 물어보았다.

10. 다른 사람들이 다르게 행동했더라면 고인의 죽음을 막을 수 있었는지 스스로에게 물어보았다.

11. 왜 이런 일이 하필 나에게 일어났는지 의문이 들었다.

12. 이 상실의 불공정함에 대해 생각해보았다.

13. 이 상실에 대한 나의 감정을 이해하려고 노력해보았다.

14. 다른 사람들이 이 상실에 대해 어떻게 반응하면 좋을지에 대해 생각해보았다.

15. 고인의 죽음을 통해 나의 삶이 어떻게 바뀌었는지 생각해보았다.

**채점 방법**

각 항목에 대한 답변은 모두 점수로 변환됩니다**. ‘한 번도 하지 않았다’**는 **1점, ‘가끔 했다’**는 **2점, ‘정기적으로 했다’**는 **3점, ‘자주 했다’**는 **4점, ‘매우 자주 했다’**는 **5점**입니다. 개별 항목에 대한 모든 점수를 합산하여 애도 반추 점수 총점을 계산합니다. 또한 이 목록은 다양한 하위 척도로 구성되어 있으므로 개별 척도의 항목 점수를 합산하여 별도로 계산할 수 있습니다. 척도 이름과 해당 항목은 다음과 같습니다.

상실의 의미와 결과에 대한 생각 1, 2, 15

사회적 지원에 관한 생각 3, 9, 14

가정형 질문 4, 8, 10

이유를 묻는 질문 5, 11, 12

감정에 대한 생각 6, 7, 13

| **Supplementary Table 1. The Cognitive-behavioral Model of COVID-related Hypochondriasis among Healthcare Workers who Witnessed Patient Deaths (N=267)** | | | | | |
| --- | --- | --- | --- | --- | --- |
| **Effect** | **Standardized**  **Estimator** | **SE** | **Z-value** | ***p*** | **95% CI** |
| **Direct effect:**  SAVE-9 → OCS | 0.22 | 0.05 | 4.63 | < 0.001 | 0.13 to 0.31 |
| **Indirect effect:**  SAVE-9 → CRBS → OCS | 0.39 | 0.04 | 9.14 | < 0.001 | 0.30 to 0.47 |
| **Total effect:**  SAVE-9 → OCS | 0.61 | 0.05 | 12.44 | < 0.001 | 0.51 to 0.70 |

Note. SE, standard error; CI, confidence interval.

OCS, Obsession with COVID-19 Scale; CRBS, Coronavirus Reassurance-seeking Behavior Scale; SAVE-9, Stress and Anxiety to Viral Epidemics-9 items

| **Supplementary Table 2. The Results of Direct, Indirect, and Total Effects on Mediation Analysis among Healthcare Workers who Witnessed Patient Deaths (N=267)** | | | | | |
| --- | --- | --- | --- | --- | --- |
| **Effect** | **Standardized**  **Estimator** | **SE** | **Z-value** | ***p*** | **95% CI** |
| **Direct effect:**  UGRS → OCS | 0.18 | 0.04 | 4.58 | < 0.001 | 0.10 to 0.26 |
| **Indirect effect:**  UGRS → SAVE-9 → OCS  UGRS → CRBS → OCS | 0.06  0.23 | 0.02  0.04 | 3.37  6.00 | < 0.001  < 0.001 | 0.03 to 0.10  0.15 to 0.30 |
| **Path coefficients**  UGRS → SAVE-9 SAVE-9 → OCS  UGRS → CRBS  CRBS → OCS | 0.33  0.19  0.39  0.58 | 0.06  0.05  0.06  0.05 | 5.63  4.21  6.87  12.35 | < 0.001  < 0.001  < 0.001  < 0.001 | 0.21 to 0.40  0.10 to 0.28  0.11 to 0.44  0.49 to 0.67 |
| **Residual covariance**  SAVE-9 ↔ CRBS | 0.48 | 0.06 | 7.89 | < 0.001 | 0.36 to 0.60 |
| **Total effect:**  UGRS → OCS | 0.47 | 0.02 | 8.69 | < 0.001 | 0.12 to 0.19 |

Note. SE, standard error; CI, confidence interval

OCS, Obsession with COVID-19 Scale; CRBS, Coronavirus Reassurance-seeking Behavior Scale; SAVE-9, Stress and Anxiety to Viral Epidemics-9 items; UGRS, Utrecht Grief Rumination Scale; PGS, Pandemic Grief Scale for healthcare workers
